# Opportunities and challenges in rheumatology research in Central Europe

**DOI:** 10.1186/s13075-017-1368-z

**Published:** 2017-09-04

**Authors:** Zoltán Szekanecz, Branimir Anic, Gábor Héjj, Iztok Holc, Aniella Hunka, Eugene Kucharz, Klaus Machold, Miroslav Mayer, Artur Pahor, Rudolf Puchner, Jozef Rovensky, Ladislav Senolt, Alena Tuchynova, Jiri Vencovsky, Josef S. Smolen

**Affiliations:** 1 Department of Rheumatology, University of Debrecen Faculty of Medicine, Nagyerdei str 98, Debrecen, H-4032 Hungary; 2 Division of Clinical Immunology and Rheumatology, Department of Internal Medicine, University of Zagreb School of Medicine, Zagreb, Croatia; 3 National Institute of Rheumatology and Physiotherapy, Budapest, Hungary; 4 Department of Rheumatology, Division of Internal Medicine, University Medical Centre of Maribor, Maribor, Slovenia; 5 Department of Internal Medicine and Rheumatology, Medical University of Silesia, Katowice, Poland; 6 Department of Rheumatology, Medical University of Vienna, Vienna, Austria; 7 Private Practice, Wels, Austria; 8 National Institute of Rheumatic Diseases, Piestany, Slovakia; 9 Institute of Rheumatology, Prague, Czech Republic

**Keywords:** Central Europe, Rheumatology, Research, Funding, Arthritis, Collaboration

## Abstract

The Central European Congress of Rheumatology (CECR) has been organized by seven Central European countries: Austria, Croatia, Czech Republic, Hungary, Poland, Slovakia, and Slovenia. These countries have lots of similarities, but also differences, with respect to rheumatology research. In this paper, based on questionnaires, we wish to demonstrate achievements and difficulties in rheumatology research performed in our region.

## Background

Central European countries have common geopolitical backgrounds and numerous similarities with respect to history and development including medical care and research. Here we will deal with the seven official organizers of the Central-Eastern Congress of Rheumatology (CECR): Austria, Croatia, Czech Republic, Hungary, Poland, Slovakia, and Slovenia. There are significant differences between the countries with respect to gross domestic product (GDP), organization of healthcare, and research funding.

In this brief review, we wish to describe the recent developments and present the current situation of clinical rheumatology and research in Central Europe. For this purpose, we sent out a questionnaire to the presidents of the national societies.

## Some indicators of rheumatology care

In order to understand rheumatological research in these countries, first we should assess postgraduate training and the definition of rheumatologists. In some countries, such as Austria and Czech Republic, most rheumatologists are also internists. On the other hand, in Hungary, about 70% of rheumatologists are not internists so many of them do not treat connective tissue diseases.

There are between 2 and 75 rheumatology hospitals/departments in these countries. Where internists treat rheumatic patients, most outpatient and inpatient care is of course performed in internal medicine units. There are variable numbers of patients treated with biologics (Table 1).

**Table 1:** Key figures in the seven countries

	Austria	Croatia	Czech Republic	Hungary	Poland	Slovakia	Slovenia
Population (millions)	8.3	4.4	10.4	10.0	38.5	5.4	2.0
GDP per capita (IMF, 2016, Int$)	47,800	22,400	33,200	27,200	27,700	31,200	32,000
No. of rheumatologists	324	47	350	800	~ 1300	71	21
No. of rheumatology departments/hospitals	10/2	18	12	31	75	1+ ***	7
No. arthritis patients on biologics	N/A	1800	5700	8500	~ 8000	2200	2200
No. of research centres (with international publications)	20	7	5	21	15	6	2
No. of research groups	20	7	13	21	15	6	2
Main research topics	• Several clinical trials in arthritides, SLE, etc • EULAR-related activities, recommendations • Registries (BIOREG), databases, biobanking • Autoantibodies • Bone/osteoimmunology • Experimental arthritis • Experimental lupus • Outcomes research (RA, PsA, SLE, OA) • Scleroderma • Ultrasonography • Osteoarthritis • Psoriatic arthritis • PMR • Health services research	• Epidemiology of SLE • Cardiovascular comorbidities • SpA • Systemic sclerosis • Osteoporosis • Osteoclast progenitors • JIA biomarkers	• Inflammatory myopathies • Biomarkers • SpA • Systemic sclerosis • Early arthritis • Osteoporosis • Urate transporters • miRNA • JIA • Registries	• Comorbidities (CV, malignancies) • RA • Systemic sclerosis • SpA • Osteoporosis • Urate transporters • Ultrasonography • Genomics • SLE • Sjögren’s • Myositis • MCTD • T and B cell subsets • Arthritis models	• RA • Pregnancy • Systemic sclerosis • Stem cell transplantation • Ultrasonography • GCA • Fibromyalgia • Immunopathology • SpA • Ped. rheumatology • Osteoporosis	• Alkaptonuria • Corticosteroids in the elderly • Hyperprolactinemia • PET-CT in vasculitis • Autoinflammatory syndromes • SLE • Osteoporosis	• Atherosclerosis in RA • Sjögren's sindrome • Vasculitis, • Adherence to treatment • Antiphospholipid syndrome
Number of publications with IF (2012–2016)	278*	90	250	315	663	55	21
National journal	*Zeitschrift für Rheumatologie* (Germany)	*Reumatizam*	*Czech Rheumatology*	*Magyar Reumatológia* (Hungarian Rheumatology)	*Reumatologia* (Rheumatology) (bimonthly; in English) *Forum Reumatologiczne* (quarterly; in Polish)	*Rheumatologia*	None
No. of PhD theses in rheumatology	4**	10	9	16	7	0	0
No. of research grants awarded	16	8	17	43	N/A	3	0

* First or last authorship

**Associate Professorship

*** Many arthritis and autoimmune patients are treated in internal medicine departments

*CV* cardiovascular, *GCA* giant cell arteritis, *GDP* gross domestic product, *IF* impact factor, *JIA* juvenile idiopathic arthritis, *MCTD* mixed connective tissue disease, *miRNA* microRNA, *N/A* not available, *OA* osteoarthritis, *PET-CT* positron emission tomography-computed tomography, *PMR* polymyalgia rheumatica, *PsA* psoriatic arthritis, *RA* rheumatoid arthritis, *SLE* systemic lupus erythematosus, *SpA* spondyloarthropathy

## Description of rheumatology research

As seen in Table 1, significant research in the field of arthritides, osteoporosis, and connective tissue diseases has been carried out over the past years. Our rheumatologists have been involved in the development of international recommendations (e.g. management of rheumatoid arthritis (RA), early RA, spondyloarthropathies, systemic sclerosis, systemic lupus erythematosus (SLE), myositis, Sjögren’s syndrome, polymyalgia rheumatica, vasculitides, osteoporosis, alkaptonuria, cardiovascular comorbidities, and ultrasound) [1–13]. There have been active members of EULAR, EUSTAR, GRAPPA, ASAS, EUROPHOSPHOLIPD, MYONET, and several other working groups. Although there have been significant limitations in funding of research, recently our countries have been involved in large European Union (EU), Horizon2020, Foreum, and other collaborative projects.

## The Central European Congress of Rheumatology

CECR congresses started in 1996 in Piestany (Slovakia), and were followed by meetings every other year in Warsaw (1998), Bratislava (2000), Baden (2002), Budapest (2004), Bled (2006), Prague (2008), Sopron (2010), Krakow (2012), Vienna (2014), and again Prague (2016). Croatia joined “the club” in 2016 and the next meeting will be in Zagreb in 2018.

## EULAR activities

The 70-year-old European League Against Rheumatism (EULAR) is the key organization in the field of rheumatology. The seven countries have nominated country representatives on the EULAR committees. In addition, Frantisek Lenoch (Czechoslovakia), Karl Gotsch (Austria), Béla Gömör (Hungary), and Josef Smolen (Austria) served as EULAR presidents. Karel Pavelka (Czech Republic) and László Czirják (Hungary) worked as General Secretary. Tadej Avcin (Slovenia) is currently Chairperson of the Standing Committee on Paediatric Rheumatology. Daniel Aletaha (Austria) chaired the Standing Committee for Clinical Affairs. Zoltan Szekanecz (Hungary) and Tadej Avcin are currently members of the Scientific Programme Committee of the EULAR congress. Past members of the Scientific Committee include Jiri Vencovsky and Karel Pavelka (Czech Republic), Wlodzimierz Maslinski (Poland), and Kurt Redlich (Austria). Jiri Vencovsky is currently acting as the treasurer for FOREUM.

The numbers of submitted and accepted EULAR abstracts are relevant indicators of the quality of rheumatology research. As listed in Table 2, the 7 countries together submitted 169, 152, 153, 133, and 138 abstracts to the last five EULAR congresses (2012–2016), respectively. The total number of submissions to these congresses was 3806, 3872, 4041, 4323, and 4109, respectively. Thus, our 7 countries submitted 4.4%, 3.9%, 3.8%, 3.1%, and 3.4% of all abstracts, respectively. The percentage of accepted abstracts (oral + poster tour + poster + abstract publication only) was 79.3%, 75.0%, 85.0%, 79.7%, and 77.5%, respectively.

**Table 2:** EULAR congress activity

	2012	2013	2014	2015	2016	Total
Submitted	169	152	153	133	138	745
Accepted	134	114	130	106	107	591
Oral	5	2	9	5	5	26
Poster tour	9	17	7	9	12	54
Poster	42	55	38	45	43	223
Publication	78	40	76	47	47	288
Acceptance rate (%)	79.3	75.0	85.0	79.7	77.5	79.3

With respect to the number of abstract submissions, in 2015 Austria, Croatia, Czech Republic, Hungary, Poland, Slovakia, and Slovenia ranked 36^th^, 48^th^, 35^th^, 30^th^, 27^th^, 51^st^, and 46^th^, respectively, among the 84 countries with abstract submissions. In 2016, these ranks were 29^th^, 53^rd^, 32^nd^, 35^th^, 28^th^, 62^nd^, and 45^th^, respectively. Austria, Croatia, Czech Republic, Hungary, Poland, Slovakia, and Slovenia submitted 150, 40, 138, 150, 190, 34, and 43 abstracts, respectively, a total of 745, to the 2012–2016 congresses. The total acceptance rates were 88.0%, 77.5%, 77.5%, 72.7%, 81.6%, 55.9%, and 88.4%, respectively. Austria (*n* = 7), Hungary (*n* = 6), Poland (*n* = 6), and Czech Republic (*n* = 5) had the most oral presentations.

The seven countries have varying population numbers (Table 1) so their relative share within the whole of Europe differs. However, the seven Central European countries took a significant share (3–4%) of abstracts submissions to recent EULAR congresses with an acceptance rate of 75–85%.

## EWRR

The first European Workshop of Rheumatology Research (EWRR) was organized in London in 1981. The idea of the annual EWRR meetings was to give a platform to mostly young researchers to present their data in basic and translational rheumatology research. To date, 37 EWRR meetings have been organized. Among our seven countries, Vienna (twice), Warsaw, Prague, and Budapest have hosted EWRR congresses. An also increasing number of presentations from Central Europe has been included in the very recent EWRR meetings.

## Problems to solve

Maybe with the exception of Austria, our countries have experienced a significant delay in the development of rheumatology research. For many years, only Austria and Czech Republic could be part of large international projects (e.g. BeTheCure, AutoCure). In many countries, research is very much centralized to the capital (Vienna, Prague, Ljubljana, Zagreb) or to nominated centres (Piestany). It is more evenly distributed among numerous university towns in Hungary and Poland. A major problem is lack of interest of young physicians in the field of rheumatology, as well as the large workload in routine clinical practice, leaving little time for research. With the advent of new therapies and novel molecular insights, this interest will hopefully rise again over the next years. Funding has been an issue and we hope that, with development, these countries could also participate in more EU- or EULAR-related projects. With respect to human resources, the "next generation" of rheumatologists is now slowly establishing itself in the international community.

## Conclusion

Although, with the exception of Austria, these countries were socialist countries, significant development has been observed in rheumatology research.

## Abbreviations

CECR: Central European Congress of Rheumatology
EU: European Union
EULAR: European League Against Rheumatism
EWRR: European Workshop of Rheumatology Research
GDP: Gross domestic product
